# Combining genetic algorithm with machine learning strategies for designing potent antimicrobial peptides

**DOI:** 10.1186/s12859-021-04156-x

**Published:** 2021-05-11

**Authors:** Kyle Boone, Cate Wisdom, Kyle Camarda, Paulette Spencer, Candan Tamerler

**Affiliations:** 1 Bioengineering Program, University of Kansas, Institute of Bioengineering Research, University of Kansas, 1530 W 15th Street, Learned Hall, Room 5109, Lawrence, KS 66045 USA; 2 Chemical and Petroleum Engineering Department, University of Kansas, 1530 West 15th Street, Learned Hall, Room 4154, Lawrence, KS 66045 USA; 3 Mechanical Engineering Department, University of Kansas, 1530 West 15th Street, Learned Hall, Room 3111, Lawrence, KS 66045 USA; 4 Institute of Bioengineering Research, University of Kansas, 1530 West 15th Street, Learned Hall, Room 3111, Lawrence, KS 66045 USA; 5 Mechanical Engineering Department, University of Kansas, 1530 W 15th St, Learned Hall, Room 3135A, Lawrence, KS 66045 USA; 6 Institute of Bioengineering Research, University of Kansas, 1530 W 15th St, Learned Hall, Room 3135A, Lawrence, KS 66045 USA

**Keywords:** Antibacterial, Antimicrobial peptide, Machine learning, Rough set theory, Genetic algorithm

## Abstract

**Background:**

Current methods in machine learning provide approaches for solving challenging, multiple constraint design problems. While deep learning and related neural networking methods have state-of-the-art performance, their vulnerability in decision making processes leading to irrational outcomes is a major concern for their implementation. With the rising antibiotic resistance, antimicrobial peptides (AMPs) have increasingly gained attention as novel therapeutic agents. This challenging design problem requires peptides which meet the multiple constraints of limiting drug-resistance in bacteria, preventing secondary infections from imbalanced microbial flora, and avoiding immune system suppression. AMPs offer a promising, bioinspired design space to targeting antimicrobial activity, but their versatility also requires the curated selection from a combinatorial sequence space. This space is too large for brute-force methods or currently known rational design approaches outside of machine learning. While there has been progress in using the design space to more effectively target AMP activity, a widely applicable approach has been elusive. The lack of transparency in machine learning has limited the advancement of scientific knowledge of how AMPs are related among each other, and the lack of general applicability for fully rational approaches has limited a broader understanding of the design space.

**Methods:**

Here we combined an evolutionary method with rough set theory, a transparent machine learning approach, for designing antimicrobial peptides (AMPs). Our method achieves the customization of AMPs using supervised learning boundaries. Our system employs in vitro bacterial assays to measure fitness, codon-representation of peptides to gain flexibility of sequence selection in DNA-space with a genetic algorithm and machine learning to further accelerate the process.

**Results:**

We use supervised machine learning and a genetic algorithm to find a peptide active against *S. epidermidis*, a common bacterial strain for implant infections, with an improved aggregation propensity average for an improved ease of synthesis.

**Conclusions:**

Our results demonstrate that AMP design can be customized to maintain activity and simplify production. To our knowledge, this is the first time when codon-based genetic algorithms combined with rough set theory methods is used for computational search on peptide sequences.

**Supplementary information:**

The online version contains supplementary material available at 10.1186/s12859-021-04156-x.

## Background

Machine learning has been a key component of the research community’s efforts to solve problems involving multiple, complex relationships ranging from the board game Go [1], facial recognition [2, 3] and protein folding [4, 5]. While neural networks are universal approximators in the sense that any correlated relationship between input and output can be found given enough data, neural networks may find correlated relationships where causation is lacking. Therefore, deep learning methods are vulnerable to making illogical connections during training. Currently, machine learning practitioners must address this vulnerability in the training process, but the lack of a complete solution for deep learning methods in the literature directs recent efforts toward Explainable Artificial Intelligence (XAI). Some machine learning methods are, by construction, less vulnerable to making illogical connections than deep learning methods. Transparency for how decisions are made is one approach in which illogical connections can be removed from the decision process. Random forest and other tree decision methods have this feature. A recent review has identified rule induction as one solution to this issue [6]. Rough set theory is a rule induction approach which tracks the ambiguity of labels to understand the strength of relationship between input and output labels. Rough set theory has been used as a data mining method for developing expertise from complex data tables [7, 8]. Logical consistency can be moved forward in machine learning by further developing rough set theory and other transparent decision approaches. A lack of transparency limits the knowledge that can be gained from neural network models. Here, we offer a transparent machine learning approach to increase the comprehension of relationships between the specific design solutions in a design space as well as to broaden the understanding of the structure of the design space beyond a single cluster of design iterations. We apply this approach to designing antimicrobial peptides (AMPs) as alternative agents to antibiotics by incorporating a rough set theory method, a transparent machine learning approach, into an evolutionary design method.

Rising antibiotic-resistant infections have become one of the growing public health concerns globally. The 2019 “Antibiotic resistance threats in the USA” report released by US Centers for Disease Control and prevention (CDC) includes the latest USA antibiotic resistance burden estimates. According to this report, more than 2.8 million antibiotic resistance infections occur in the USA each year. The World Health Organization acknowledges the current problem of drug resistance through the Global Action Plan for AMR in 2014 [9]. The report provides urgent threats such as carbapenem-resistant *Acinetobacter*, vancomycin-resistant *Enterococcus*, methicillin-resistant *Staphylococcus aureus*, erythromycin and clindamycin-resistant *Streptococcus*. Even for current antibiotics that are being rationed for last resort, bacterial resistance is spreading quickly and widely. As an example, resistance to a polymyxin, called colistin, has spread from animals to humans in China through food chain supply [10, 11]. Beyond the declining efficacy because of the indiscriminate use of antibiotics, these drugs also lead to personal health issues such as the dysregulation of microbial communities and patient immune system suppression. The prevention of immune system dysbiosis, as in atopic march [12], from antibiotics is a further benefit of targeting antimicrobial activity. Alternative antimicrobial agents which are as effective and biocompatible as natural immune system components have become an urgent need.

Antimicrobial peptides (AMPs) have been increasingly gaining attention as new antimicrobial agents alternative to antibiotics. AMPs are essential components of innate immune systems of all multi-cellular organisms fighting as the first line defenders against a foreign attack [13–15]. Compared to conventional antibiotics, AMPs have a wide range of antimicrobial mechanisms including disruption the integrity of the bacterial membrane as well as the inhibition of DNA, RNA, and protein synthesis of the invading pathogens, or inactivating intracellular enzymes or disrupting cell wall synthesis [16–18]. With their broad spectrum as well as targeted antimicrobial efficacy, they offer an opportunity to treat even antibiotic resistant microbes [19]. We have demonstrated that these peptides can be designed for their local delivery on implantable materials as well as integrated into adhesives between materials-tissue interfaces to prevent bacteria viability on implants [20–26].

AMPs display remarkable structural and functional diversity with a massive number of possible peptide sequences, with known examples of multiple active structures for a single AMP [27, 28]. More than 2800 peptides have been isolated from a wide range of organisms [29, 30]. To expand this class of new antimicrobial agents, antibacterial peptide-mimics are introduced as another source to the existing peptide libraries as well as computational methods have been integrated into this search to find many more candidates [31–33].

While a recent study has demonstrated a narrow example of rationally designed antimicrobial peptides for targeted antimicrobial activity against *Enterococcus faecalis*,[34] no method has been developed for using broadly applicable physicochemical properties to design antimicrobial peptides for addressing a range of activity targets. Two main approaches exist in the literature for designing antimicrobial peptides. The first approach is to design new peptides rationally through curated insights into antimicrobial activity. The Joker algorithm is a recent example of inserting patterns into sequences to produce new antimicrobial peptide sequences rapidly [35]. The second main approach is through opaque machine learning methods which leverage trends in sequence data but do not yield curated insights for further exploration. Deep-neural networks describing antimicrobial sequences use this approach [36–39]. A recent study has designed antimicrobial peptides through an evolutionary algorithm [18]. While this study does provide insight into more effective peptide designs through amino acid substitution frequencies, the study did not find useful relationships through physicochemical properties. We provide a machine learning approach which transparently selects physicochemical features within the given knowledge domain in a non-linear way; this method leverages trends in datasets too large to analyze manually to provide an automated approach for rationally designing antimicrobial peptides.

Computer-aided molecular design (CAMD) is a framework for designing new functional molecules from quantitative models of activity. CAMD combines quantitative approaches of describing molecular structure and their activities in the forward problem, but also introduces the reverse problem of using these relationships to design novel molecules to meet specific design targets through intelligent search [40–42]. In the past two decades, the main approach applied to the forward problem of CAMD for antimicrobial peptides has been neural network models. In 2011, a study using improved cheminformatics descriptors reported a 94% true positive rate when synthesizing the top-fifty predicted antimicrobial peptides [43]. More recent studies have taken advantage of deep neural network architecture for the semantic language performance and addressed the importance of how to encode amino acids numerically [44–46].

In our prior art, we pioneered the use of rough set theory for the classification of peptide sequences according to antibacterial activity [47]. Our rough set theory method provides a transparent selection approach which provides explicit boundaries between physicochemical properties that active sequences possess and inactive sequences do not possess. The more boundaries which a peptide fits in with active peptides, the more likely the peptide is to be robust with different mechanism of action. Because our method produces explicit decision components, we can test sequences which contain multiple components.

**Fig. 1:**
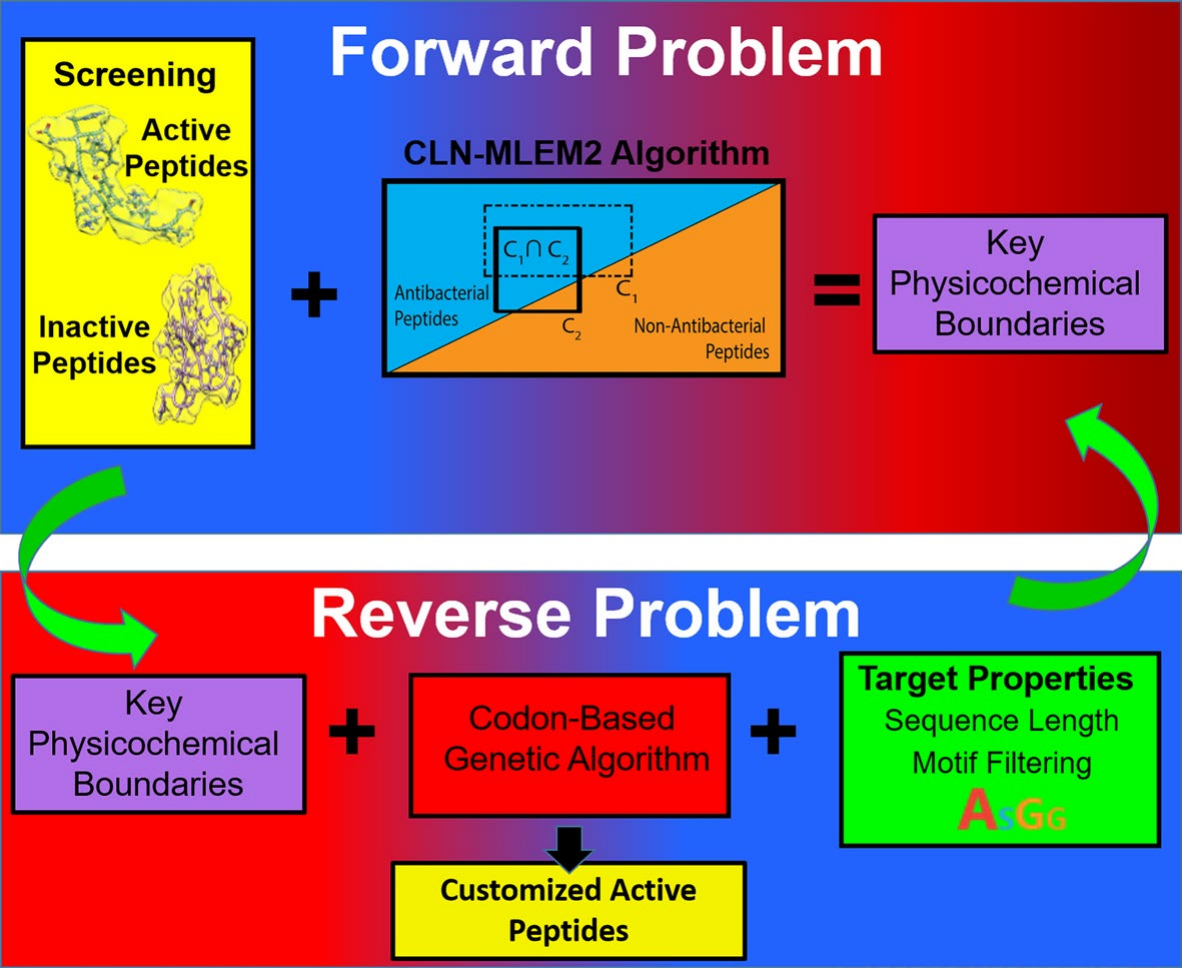
Computer aided molecular design. CAMD is an iterative two-step approach to design new antimicrobial peptides. The first step, the forward problem, is to determine quantitative relationships between antimicrobial peptide sequence and antibacterial activity. The second step, the reverse problem, is to find new antimicrobial peptide sequences with desired properties, e.g. improved ease of synthesis, through an intelligent search method. Adapted from [47]

In this paper, for the first time in a CAMD approach, we combine a rough set theory method with a genetic algorithm search to tailor antimicrobial peptides for targeted properties. For the first time in a genetic algorithm approach to designing peptides, a codon-basis will be used to increase the variation of peptide sequences generated for this intelligent search. The codon-based genetic algorithm (CB-GA) search completes the reverse problem of CAMD (Fig. 1). We demonstrate our novel CAMD approach by designing antimicrobial peptides which are targeted against *S. epidermidis* and for the ease of solid-state peptide synthesis. Our approach combines in vitro bacterial assays as the AMP fitness, genetic algorithm to uncover diversity for customizing the design through a codon-representation of peptides to direct the selection of sequences related in DNA-space, and transparent machine learning to guide the sequence selection process. Our results demonstrate that antimicrobial peptide design can be customized to maintain activity and simplify production. The proposed approach could be extended to peptide design with other desirable activities.

## Results

### Generation of increased sequence diversity through codon-basis

**Fig. 2:**
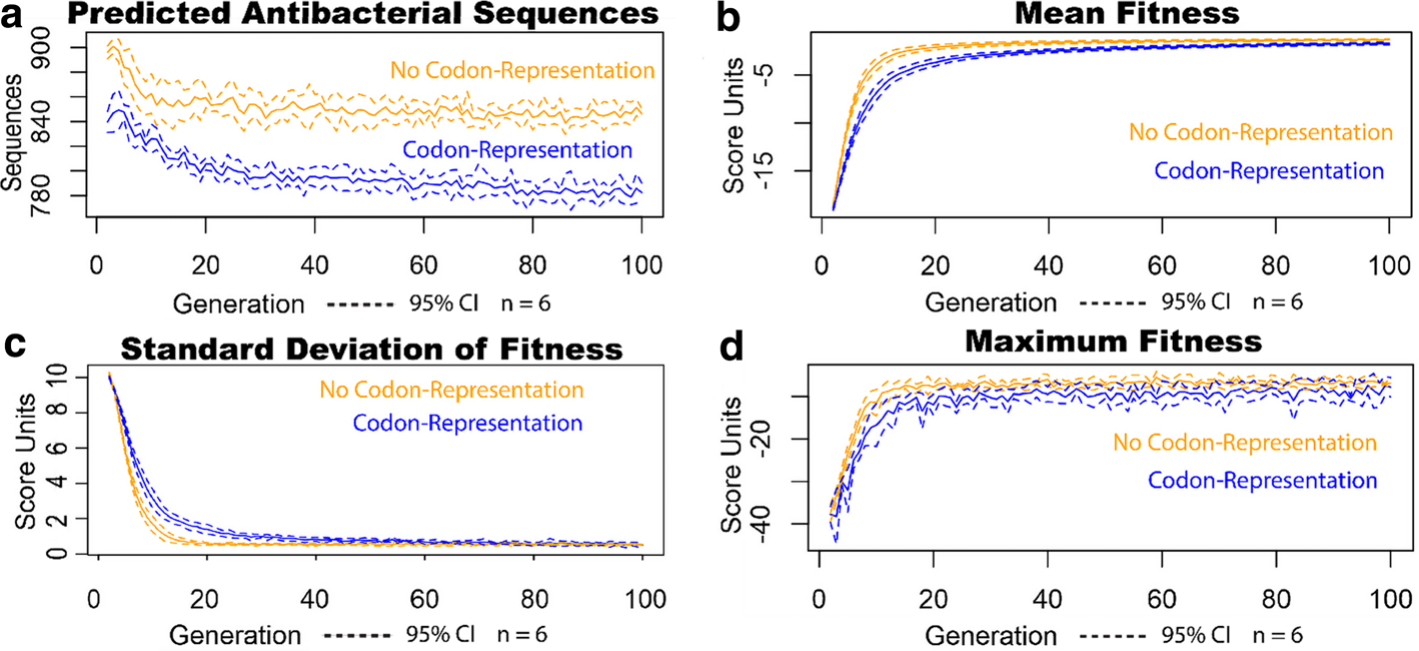
Codon-representation method increases scoring variation in beginning generations of algorithm runs. The blue lines represent data for the codon-representation and the orange lines represent data without the codon-representation. The solid lines represent the average of 6 repeated genetic runs over 100 generations. The dotted lines represent the 95% CI using the student t test statistic of the repeated runs. **a** The number of predicted antibacterial sequences decreases when using the codon representation, **b** While the beginning mean fitness of the codon-representation is worse, the mean fitness converges for both methods due to the filtering of each pool by the top-scoring sequences, **c** The beginning standard deviation of fitness scores is higher with codon representation, but it converges with the non-codon representation by the sequence score filtering. **d** The maximum fitness also shows a similar trend as mean fitness, showing that the best scoring sequences for either method converge to similar scores. The increased scoring variation likely comes from a wider parameter space coverage. An advantage of using codon representation is the increased parameter coverage for screened peptides

Motivated by aggressive mutations that codon representation can offer, we convert the peptide sequences to a codon-representation to take advantage of reading frames for generating novel antimicrobial peptide sequences. We mutate a single DNA base through substitution, insertion or deletion and search for novel sequences through cross-over by combining subsequences of predicted antimicrobial peptides. The antimicrobial peptide amino acid frequency to codon frequency ratio is given in Fig S1, showing that most amino acid frequencies are close to their codon frequencies in the standard codon table. Figure 2 shows that using the codon-representation increases the variance of generated fitness scores at the beginning of the generations, while reaching similar score variance, maximum and mean fitness levels as without codon-representation. Filtering by top-scoring sequences reduces the variance of scores for both methods. The results shown are for single-threaded genetic algorithm runs. Our approach allows for completing multiple trajectories from the clustering of sequences in previous generations.

The number of predicted antibacterial sequences are screened from the number of sequences initially generated by the genetic algorithm. Only the sequences which adhere to the MLEM2 rules for antibacterial activity pass this screening. Use of the codon-representation results in less generated peptide sequences passing this screen (Fig. 2a). However, the codon-representation also results in more varied fitness scores for screened sequences as seen in Fig. 2c. We further extended our analyses to compare the codon-representation to non-codon representation with respect to the degree of variability of selection of these sequences.

**Fig. 3:**
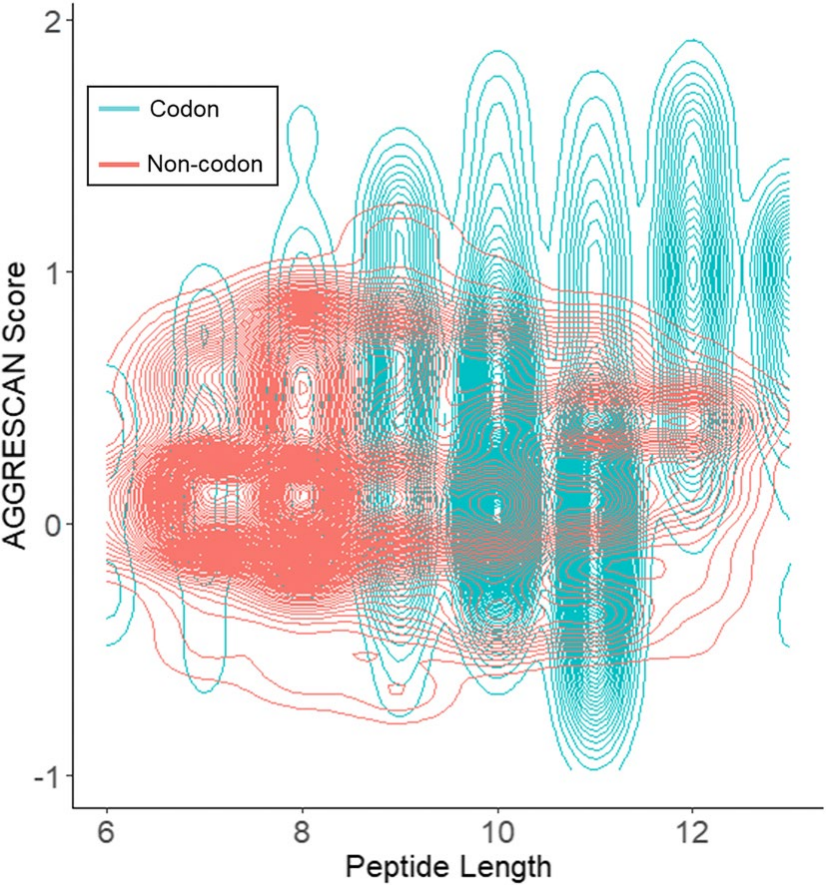
Widened parameter coverage for screened peptides through codon-representation. Generation 20 was shown to have higher scoring variation for codon-representation peptides in Fig. 4c. The spread of the peptides predicted to be antibacterial either generated by the codon representation method (teal) or the non-codon representation method (red) is shown. Each contour is a change in density of 0.01. The 2-D density contours show that the codon-representation generates screened peptide sequences which have AGGRESCAN scores outside the non-codon representation at most peptide lengths, both above and below, even though the non-codon representation generated more screened sequences as seen in Fig. 4a. The codon-representation also generated the majority of peptides at the extremes of peptide length, 6 and 13

The codon-representation peptide generation method was evaluated if its peptides screened to be antibacterial by our CLN-MLEM2 rules were more diverse than without the codon-representation. In Fig. 3, we plotted the contour map of parameter coverage for each approach for two parameters of our scoring function, by peptide length and AGGRESCAN score. Crossing each contour changes the 2-D density estimation of screened peptides generated by the given method in the nearby parameter area by one percent. We selected the 20^th^ generation as an early generation in which the scoring standard deviation was still elevated when using codons.

Figure 3 shows that the codon representation results in better coverage of the parameter space for screened sequences due to the areas covered by the codon representation with no coverage by the non-codon representation. The increase in coverage in Fig. 3 is expected because the non-codon method is a subroutine of the codon method when there are no reading frame shifts. Such a shift will occur with odds of 2:1 because for every three DNA positions to select as a starting position, one results in no frame shift and two result in frame shifts. For codons of arbitrary length, the increase in the codon length would increase the odds of frame shifts. Moving from the natural 3-base codon table to a 4-base codon table would result in an increase of frame shift probability by a factor of 3/2. If such an increase in frameshifts results in better genetic algorithm performance is the domain of future study.

Our methodology of advancing our search by generation with frameshift sequences increases our sequence variability but may reduce our average sequence scores. This increased access in parameter space was evaluated for a potential tradeoff of not finding peptides which are comparatively highly scoring within the same number of generations. As the generation number increased for both methods, the coverage of the parameter space narrowed due to the filtering of the top-scoring sequences, seen in Fig. 2c. We did not see a loss in the mean score reached or in a reproducible loss in the maximum score reached for algorithm runs of 100 generations, as seen in Fig. 2b and 2d respectively. We get access to predicted active sequences which have a wider range of parameter values. Although our results were plotted with two dimensions to show the widened coverage, our genetic algorithm optimization considers parameters in many dimensions at once.

### Novel antimicrobial peptide generation

**Fig. 4:**
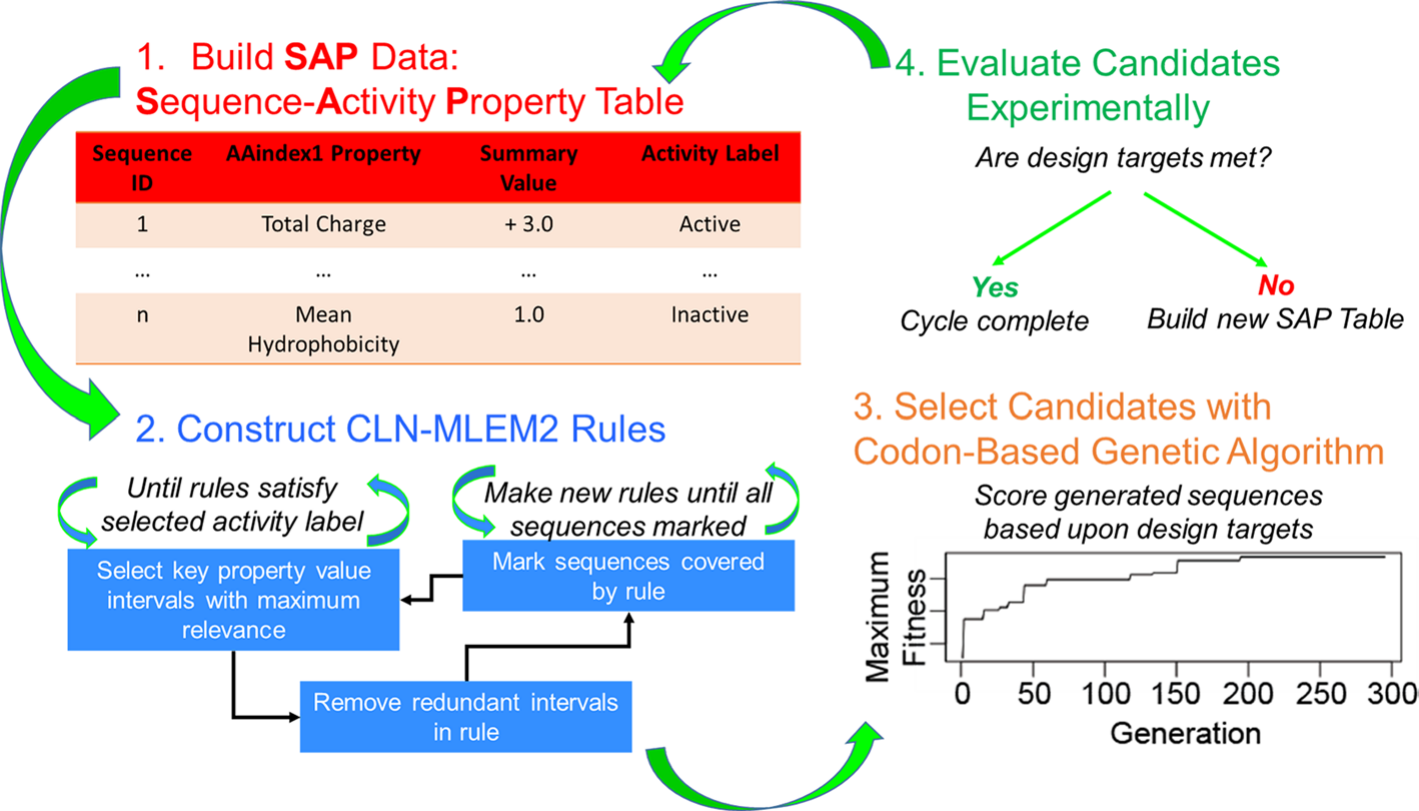
Customized active peptide design method. Steps 1–3 induct rules which separate active from inactive peptides. Step 4 finds new sequences which are predicted to be active and are customized toward design targets. The peptides are evaluated for activity. If new sequences are active, then the process is complete. If the new sequences are inactive, the method starts a new iteration by learning which new sequences to test by incorporating the previously customized sequences in the dataset

We previously published our solution to the forward problem of CAMD antimicrobial peptide design [47]. Here, we describe our solution to the reverse problem of CAMD antimicrobial peptide design with our Codon-Based Genetic Algorithm (CB-GA) method (Fig. 4). We start the first generation from known antimicrobial peptides from the APD3 [29] and sort them by score using our design targets. For this work, we target peptide sequences which are easily synthesizable with the fluorenylmethoxycarbonyl (FMOC) protection method (Table 1). Our scoring function for fitness is the negative weighted average of the distance from the targets. We targeted relatively short amino acid sequences because shorter sequences are faster and cheaper to synthesize. Cysteine residues add to the complexity of the synthesis process by introducing inter-peptide bonding and intra-peptide bonding between residues through disulfide bonds. We have simplified this level of complexity by selecting sequences which do not have cysteine. Another consideration of working with the synthesized peptides is their stability in solution. This property can be estimated to a first approximation with the likelihood that the peptide sequence will aggregate to itself. We use the Aggrescan method to make this prediction, with lower numbers leading to a lower chance of aggregation [48]. Having peptides which are less likely to aggregate may select for peptides which are not as permeable to bacteria membranes. To compensate for this possible loss of activity while keeping the lower aggregation property, we added the net positive charge target property to restrict our search to cationic peptide sequences, which are among the commonly kown active examples [29, 49].

**Table 1:** Design targets for novel antimicrobial peptides for ease of FMOC synthesis

Property	Target
Amino acid length	7 to 15
AGGRESCAN score	− 1.25
Cysteine count	0
Net positive charge	+ 1 to + 5
Matching CLN-MLEM2 rule count	8–12

**Fig. 5:**
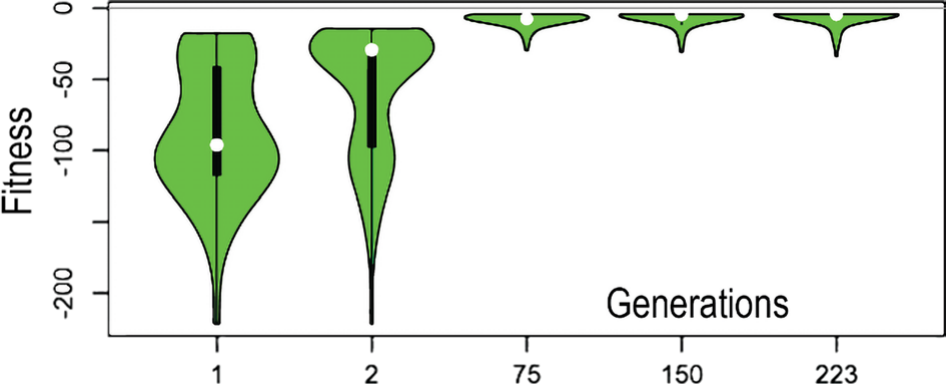
Fitness distributions of selected generations. Fitness score distribution across selected generations as violin plots. The center of the violin plot is a box plot and the sides of the violin plot are the probability density kernels. The gray line is the score of zero, a perfect score representing all design targets being met. Generations are sequentially produced until a limit of non-improving generations is reached. A generation is non-improving if none of the members of the generation have an improved fitness score from the previous generations

Meeting multiple CLN-MLEM2 rule categories likely increases the probability of peptide activity by having multiple features which are selective for being antibacterial. Therefore, we use the MLEM2 rule category count as a design target (Table 1). We hypothesize that peptide sequences with different distinguishing descriptions of activity may have multiple, distinct mechanisms of action. We observed genetic algorithm improvement toward our design targets across generations to improve meeting our design targets which combine MLEM2 rule categories (Fig. 5).

The newly generated sequences are filtered by our high specificity rough set theory classification method CLN-MLEM2 [47] so that each completed generation only consists of either known antimicrobial peptides from the initial generation or predicted antimicrobial peptides. Additional files 1, 2 contains the pool of generated sequences. Violin plots [50] of the fitness scores, where the center is a boxplot and the shape of the sides is formed from the probability density kernel, across selected generations is shown in Fig. 5. Advancing generations become more skewed in favor of higher fitness scores.

The antibacterial screening with *S. epidermidis* showed that one of the three antimicrobial peptides selected from our genetic algorithm showed antibacterial activity. As a positive control we include Hp1404 [51], the highest scoring peptide from our initial peptide pool from APD3—AMP database (APD3: Antimicrobial peptide calculator and predictor). Hp1404 has known activity against gram positive bacteria. The AMP-2 sequence’s aggregation potential sequence average (-0.02) is reduced compared to Hp1404 (0.26), and the AMP-2 peptide still retains activity against S. epidermidis. The average aggregation potential for AMP-2 is at the hot-spot threshold [48]. The middle of the Hp1404 sequence was conserved among novel peptides for this genetic algorithm search. Conserved residues are underlined in Table 2. Close sequence similarity relationships between AMP-1 and AMP-2 did not result in similar antibacterial activity against *S. epidermidis*. While AMP-2 and AMP-3 also share conserved residues, their activities were varied. New MLEM2 rule categories can be generated to discriminate sequences with these differences for future iterations.

**Table 2:** Screened antimicrobial activities against S. epidermidis by the candidate antimicrobial peptides by the MLEM2 rule categories (AMP-1, AMP-2, AMP-3) and positive AMP control, Hp1404. Underlined letters indicate residues conserved in novel peptides compared to Hp1404

Agent	Sequence	Concentration (mg/mL)	Inhibition zone (cm)
Ampicillin	n/a	0.01	1.6
Hp1404 (crude)	**G**I**LG**K**LWEGV**KSIF	4.00	2.3
AMP-1 (crude)	AT**LG**V**LWE**SIRGHR	4.00	0
AMP-2 (crude)	AT**LG**V**LWEG**ARGHT	4.00	1.2
AMP-3 (crude)	**G**T**L**ANG**WEGV**RTNH	4.00	0

We attempted a translated basic-local alignment search tool with nucleotides (tblastn) on its public server [52] for the designed peptide sequences against the National Center for Biotechnology Information (NCBI). Also RefSeq Genome search was carried by different substitution matrices (BLOSUM45, BLOSUM62, BLOSUM90) with the lowest gap penalties and high expected scores (0.05, 0.1 and 0.25). The designed peptides were not found to have high homology with encrypted antimicrobial peptides from known DNA sequences.[53]

## Discussions

### Increasing variation through codon representation

Genetic algorithms have been used to design molecules of targeted properties for a variety of problems [54, 55]. Protein or peptide design is a natural application of genetic algorithms since the basic algorithm was inspired by natural protein evolution. Computational protein and peptide design has been accomplished through genetic algorithms [56–62] before but not all aspects of the genetic system that inspired genetic algorithms have been explored in protein and peptide design. We apply a DNA codon representation of peptides within our genetic algorithm to take advantage of reading frameshifts.

Generating novel solutions in a genetic algorithm is a balance between viability, finding solutions that meet some criteria, and adaptability, finding solutions that meet all criteria. Increasing the viability of each generation often involves using small moves in sequence space to avoid the loss of viability of large, random moves. Increasing the adaptability relies on the ability to make bigger moves in sequence space to preserve genetic diversity among generations. Reading frameshifts in biology represent one of these large moves that balances viability and adaptability as biological proteins develop in nature. Single-codon mutations in DNA, either deletions or insertions, cause reading frameshifts. Reading frameshifts encode transition probabilities for which new amino acids replace the previous amino acids. While most-reading frameshifts are nonviable, the viable frame shifts in nature may lead to the gain-of-function mutations. While using a codon-representation reduces the viability of our method by generating less predicted antibacterial sequences (Fig. 2a), the generation diversity is increased, as seen by the increase of the spread of scores (Fig. 2c) and in the wider coverage of target score dimensions (Fig. 3). Since our method filters out non-antibacterial sequences, this increased generation diversity is among predicted antimicrobial peptides.

To the authors’ knowledge, this is the first time a genetic algorithm to design peptides [43, 63–71] has used reading frameshifts for generating novel sequences. While the codon representation is a component of natural protein evolution, we do not believe that this is sufficient evidence that the natural codon representation is suited for the de novo designing peptides of a targeted activity. We are investigating how shifting reading frames in certain codon representations may yield low-dimensional spaces in which neutral or gain-of-function mutations may become accessible. We start with a natural codon table to benchmark the peptide generating properties which fit our rough set theory predictions. Future work will address how changes in the codon table affect the generating peptide properties for a targeted activity.

### Combining antibacterial classes

Each CLN-MLEM2 rule for antibacterial activity describes a set of physicochemical properties that separates a set of antibacterial peptides from all given non-antibacterial peptides in the training set. Some peptides may meet more than one rule for antibacterial activity. These peptides may act in multiple ways to achieve antibacterial activity. Measuring the number of CLN-MLEM2 rules a peptide meets may be a measure of its robustness for having broad spectrum antibacterial activity because the different rules might represent different mechanisms of activity. Combining these sequence features may also combine the different mechanisms of activity.

## Conclusions

Machine learning is accelerating many important design problems such as the design of antimicrobial peptide sequences to combat drug resistance in bacteria and to reduce antibiotic suppression of the host immune system. The transparency of the machine learning algorithm can be used to gain better comprehension of important design problems. In this paper, we offer a transparent machine learning algorithm, rough set theory, combined with an evolutionary search method with improved sequence diversity generation to customize antimicrobial peptides for simplified manufacturing while maintaining their activity. To improve the targeting of antimicrobial activity to address antibiotic drug resistance, microbiome dysbiosis and immune system suppression simultaneously, our proposed computer-aided molecular design (CAMD) approach allows to design antimicrobial peptides with targeted desired properties and strain specificity. We demonstrated that our method found novel antibacterial peptides that are easier to synthesize than antimicrobial peptides in the APD3 database. We also improved the antibacterial activity with more novel antimicrobial peptides by adding together multiple rules for activity from our rough set theory method. For the forward problem of quantifying sequence-activity relationships, we applied our rough set theory method (CLN-MLEM2) as a quantitative structure–activity relationship (QSAR) model to designing peptides. For the reverse problem of finding novel peptide sequences, we applied our codon-based genetic algorithm to discover novel antibacterial sequences against *S. epidermidis*, a key pathogen for implant infections. Our in vitro activity results for *S. epidermidis* support the transparent machine learning approach that can be expanded to include different pathogens. Overall, the developed codon based genetic algorithm technique offers sequence diversity, combined with rough set theory methods can be used for generating novel peptides with targeted properties.

## Methods

### Rough set-based active peptide customization method

Rough set theory[72] is a heuristic method for finding the most relevant property value intervals which differ between classification labels. We have shown that intervals of summary sequence property values calculated from amino acid chemical properties in the AAindex1[73] can be used to separate active peptide sequences from inactive sequences [47]. The summary sequence functions are described in Table S1. The chemical property intervals are described as conditions in the context of rough set theory. A rule is the intersected set of conditions such that the set only has a single class label for each of its members. In complex datasets, even using all properties available in a dataset may not result in a set with only one selected class label. Using a relaxed criterion for discernibility such as modified learning from experience module 2 (MLEM2) rules [74], a rule may still be acceptable if a certain proportion, called α, corresponds to the single, selected class. If not, the rule is considered vague and removed from the rule set. Rules which use large numbers of properties may be at greater risk of overfitting. We developed a variation of MLEM2 which limits the number of conditions in the generated rules. Since conditions are collections of property values, limiting the conditions is expected to reduce the number of properties selected by the rules. The CLN-MLEM2 (Condition-Limited Number Modified Learning from Experience Module 2) was shown to have high specificity performance when classifying antimicrobial peptides from inactive peptides. We use rough set theory to create rules which separate active from inactive peptide sequences. The AAindex1 properties and their descriptions selected by the CLN-MLEM2 rule method are listed in Table S2. The selection distribution of AAindex1 properties among CLN-MLEM2 rules is given in Figure S1. Properties relating to alpha-helix formation and for coil formation appear the most frequently in our rules. See the first three steps of Fig. 4. The fourth step is to predict new active sequences. Once these sequences are evaluated, they can be added into the dataset, as the step between Step 4 of the current iteration and Step 1 of the next iteration. Updated rules are generated for each iteration.

### Initial datasets

For CLN-MLEM2 rule generation, the positive training dataset was the S1 set (“Antibacterial”) from iAMP-2L, which has 1,274 unique sequences [75]. The negative training set of data was the non-AMP dataset from iAMP-2L, which has 1,440 unique sequences [75].

For the codon-based genetic algorithm, the initial dataset was the positive antimicrobial peptide set for the initial generation from the APD3 Antimicrobial Peptide Database.

### Codon-based genetic algorithm for finding customized peptide sequences

The genetic algorithm begins by ranking known antimicrobial peptides according to a given set of design targets as seen in Fig. 3. The initial step begins with a set of antimicrobial peptides. The next step is to rank the peptides according to the design targets. The top 25% of scoring candidates are selected to mutate and crossover by a DNA codon representation to generate novel peptide sequences. For amino acids represented by multiple codons, the representative DNA codon is uniformly selected among these codons. If all scoring candidates are copied between generations, the number of candidates grows exponentially. While removing the bottom 75% reduces the genetic diversity of future generations, it improves the convergence of the solutions to find new sequences with less computation time. The diversity lost with the filtering of the top candidates is partially replaced by recombination operators. Fig. S2 provides the expected AMP amino acid frequency by codon count.

To minimize the computational time to find new antimicrobial peptide solutions, we first filter by retaining only unique sequences from the generated sequence pool once the recombination operations are finished. Secondly, we filter the novel sequences by the antimicrobial peptide classifier. The sequences remaining in each generation are both unique and predicted to be antimicrobial by our MLEM2 method. These two steps also limit the exponential growth of the candidate pool. Partially redundant sequences are indications of patterns that may be useful to include when generating new sequences, provided that these patterns are selective for being active. These partial redundancies are referred to as motifs. Many of these motifs can be captured through generating MLEM2 rules to describe active or inactive key physicochemical properties when they are distinct for activity. The repeating patterns which result in matching MLEM2 rules will dominate the newly generated sequences to arrive at a locally optimized solution. Since the best sequences are copied to the next generation, the highest scoring sequence across generations is in the final generation.

**Fig. 6:**
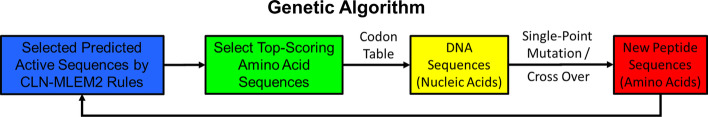
Codon-based genetic algorithm. The overall steps for producing each generation are shown in (**a**) and the steps for using a codon table to produce novel sequences is shown in (**b**)

The genetic algorithm implementation in this work gains flexibility in the moves it considers by using a codon-representation of peptides to direct the selection of sequences related in DNA-space (Fig. 6). The process of peptide sequence conversion to DNA codons is the reverse of the information flow which occur in transcription and translation processes in biology [76]. The information flow of the processes of transcription and translation of mapping nucleic acid sequences to amino acids are applied to the DNA codon representation to recover the peptide sequence following the mutation and crossover events. Small moves in the DNA-space might be large moves in the protein sequence space due to reading frameshifts. Integrating the codon table concept generates novel sequences to take advantage of the transition probabilities encoded in reading frameshifts. Reading frameshifts are changes to the nucleic acid base position which results in different codons being read downstream in the nucleic acid code. The DNA code of … “ATGATG” … would result in the amino acid code … “Met-Met” … if read from the first letter or as … “STOP” – ending the transcription, if read from the second letter. To direct the genetic algorithm toward feasible answers, the highest scoring sequences are copied between generations. Making new candidates instead of modifying current candidate sequences builds in a historical property such that the best old sequences are propagated to future generations if they are competitive with the newly generated sequences.

### Peptide synthesis

Peptides were chemically synthesized using an Aapptec Focus XC peptide synthesizer. The peptide-resins were assembled on Wang resins with C-terminal amino acids using FMOC chemistry. The N-terminal Fmoc deprotection was performed by treatment with 20% piperidine/dimethylformamide (DMF) in a 0.2 mmol reaction scale with mixing and nitrogen gas bubbling. Effective removal of the Fmoc protecting group was monitored by UV spectroscopy. The peptide-resin was filtered, and the 20% piperidine/DMF solution was added repeatedly until complete deprotection quantified by UV spectroscopy. Typically, two cycles of deprotection were sufficient. The peptide-resins were then washed with DMF. Activation of 0.2 M amino acids/DMF (2 equivalent to reaction scale) was performed by addition of 0.2 M 2-(1H-benzotriazol-1-yl)-1,1,3,3-tetramethyluronium hexafluorophosphate (HBTU)/DMF (2 equiv.) in a measuring vessel then added to the reaction vessel containing the deprotected peptide-resin for a 45-min coupling reaction. The coupling step was completed twice to ensure addition of the desired amino acid. The procedure was repeated until the complete peptide was assembled on the solid resin support. Following synthesis, the peptide-resin was removed from the reaction vessel using DMF. DMF was removed from the peptide-resin by washing with ethanol and drying on a coarse-grained glass fritted Buchner funnel. The dried resin was transferred to a glass volumetric flask followed by addition of a cleavage cocktail (15 mL/ 1 g of resin) for two hours with gentle stirring to remove the peptide from the solid support and remove the side chain protecting groups. The standard cleavage cocktail was trifluoroacetic acid (TFA)/triisopropylsilane (TIS) / water (95:2.5:2.5, % vol/vol/vol). To remove side chain protecting groups from peptides containing histidine or cysteine, 2.5% thioanisole and 2.5% 1,2 ethanedithiol were added to the cocktail and for peptides containing methionine, tyrosine, or arginine, 5% phenol was added. The cleavage products were filtered in a glass Buchner funnel and crude peptide product was isolated by precipitation in cold ether (30 mL). The crude peptide was pelleted by centrifugation (2000 rpm for 2 min), the supernatant was removed, the pellet was resuspended in ether and recentrifuged for a total of four times. Following ether washes the crude peptide products were lyophilized. The mass spectra of the synthesized peptides are provided in Figs S3-S6.

### Zone of inhibition tests

*Staphylococcus epidermidis* ATCC® 29886TM was used in the present study. The strain was cultured according to ATCC® protocol using the Nutrient agar (Difco 0001) or Nutrient Broth (NB) (Difco 0003). The bacterial pellet obtained from ATCC was rehydrated in 0.5 mL of the above-specified media, and several drops of the suspension were immediately placed and streaked on an agar slant of the specified media. The agar-plate was then incubated aerobically at 37 °C for 24 h. Overnight cultures of *S. epidermidis* were made by aseptically transferring a single colony forming unit into 10 mL of NB, followed by aerobic incubation at 37 °C with constant agitation (200 rpm) for 16 h.

AMP functional peptide candidates were screened for antimicrobial function using a zone of inhibition assay on agar plates. Crude peptides were dissolved in dimethyl sulfoxide (DMSO)/H_2_O. The bacterial growth culture was spread on agar plates using a sterile cotton swab then 10µL of the solubilized peptide candidates were pipetted in triplicate on the bacteria coated agar and incubated 24 h at 37 °C, 5% CO_2_. Plates were removed from the incubator and the zone of inhibition of bacterial growth were photographed and measured. 10 µg/mL ampicillin was used as a positive control and 2% DMSO/ H_2_O as a negative control.

## Supplementary information

**Additional file 1.** Supporting information.

**Additional file 2.** Sequence pools.

## Data Availability

The datasets used and/or analyzed during the current study are available from the corresponding author on reasonable request.

## Abbreviations

AAindex1: Amino acid index 1
APD3: Antimicrobial peptide database 3
AMP: Antimicrobial peptide
CAMD: Computer aided molecular design
CB-GA: Codon-based genetic algorithm
CDC: Centers for disease control and prevention
CLN: Condition-limit number
DMF: Dimethylformamide
DMSO: Dimethyl sulfoxide
FMOC: Fluorenylmethoxycarbonyl
HBTU: 2-(1H-benzotriazol-1-yl)-1,1,3,3-tetramethyluronium hexafluorophosphate
MLEM2: Modified Learning from Experience Module 2
NB: Nutrient broth
NCBI: National Center for Biotechnology Information
tblastn: Translated basic-local alignment search tool with nucleotides
TFA: Trifluoroacetic acid
TIS: Triisopropylsilane
XAI: Explainable artificial intelligence
