# Supplementary material for: Combining genetic algorithm with machine learning strategies for designing potent antimicrobial peptides
Source: BMC Bioinformatics. 2021 May 11;22:239. doi: 10.1186/s12859-021-04156-x (PMC8111958; doi:10.1186/s12859-021-04156-x)
Supplement: Supplementary file 1 — Additional file 1. Supporting information. [file 12859_2021_4156_MOESM1_ESM.docx]

Supporting Information: Combining Genetic Algorithm with Machine Learning Strategies for Designing Potent Antimicrobial Peptides

Authors:

Kyle Boone

1530 W 15th Street

Learned Hall, Room 5109

Lawrence, KS 66045

Bioengineering Program, University of Kansas

Institute of Bioengineering Research, University of Kansas

[kyle.boone@ku.edu](mailto:kyle.boone@ku.edu)

Cate Wisdom

1530 W 15th Street

Lawrence, KS 66045

Bioengineering Program, University of Kansas

[cate.wisdom@ku.edu](mailto:cate.wisdom@ku.edu)

Kyle Camarda

1530 West 15th Street

Learned Hall, Room 4154

Lawrence, KS 66045

Chemical and Petroleum Engineering Department, University of Kansas

[camarda@ku.edu](mailto:camarda@ku.edu)

Paulette Spencer

1530 West 15th Street

Learned Hall, Room 3111

Lawrence, KS 66045

Mechanical Engineering Department, University of Kansas

Institute of Bioengineering Research, University of Kansas

[pspencer@ku.edu](mailto:pspencer@ku.edu)

Candan Tamerler (corresponding author)*

1530 W 15th St

Learned Hall, Room 3135A

Lawrence, KS 66045

Mechanical Engineering Department, University of Kansas

Institute of Bioengineering Research, University of Kansas

[ctamerler@ku.edu](mailto:ctamerler@ku.edu)

## Rough Set Theory Analysis

We take a data mining approach with rough set theory to understand how to describe antibacterial activity in terms of amino acid properties. For defining active and inactive sequences, we used training sets from the published iAMP-2L dataset.[1] The positive training set consisted of 1,274 sequences which originated from APD2[2] and the negative training set consisted of 1,440 sequences which originated from intracellular protein sequences from UniProt. To select from amino acid physicochemical properties, we used the AAindex1[3] as implemented in the ‘seqinr’ package in R.[4] As previously published,[5] we selected non-correlated features to describe the amino acids in the sequences. We selected a correlation cut-off of 0.6, leaving us with 74 AAindex1 features from which to select. We describe the overall properties of the sequences by one of three mathematical operations: sum, mean or window. These operations are described in Table S1. The CLN-MLEM2 method selected from among 74 AAindex1 properties x 3 operations = 222 features. CLN-MLEM2 is an embedded feature selection method with the limit of 8 conditions per rule was set. Only 19 of the 74 AAindex properties (25.7%) are represented in the rules generated. These AAindex1 properties are listed in Table S2. 46 of the possible 222 features (20.7%) are represented in the rules generated. While many of the 19 AAindex1 properties in Table S2 have previously been identified as of interest in relating antimicrobial peptide activity,[6, 7] several novel AAindex1 properties relating to antibacterial activity have been identified with the CLN-MLEM2 method such as QIAN880139 and ROBB760107.

**Table S1: Description of summary functions used as input for the CLN-MLEM2 method for generating chemical property features.**

| **Summary Function** | **Description** |
| --- | --- |
| Sum | $\sum a_{i}$, where *a_i_* is the amino acid chemical property at position *i* from 1 to *n*, the length of the peptide sequence |
| Mean | $\sum\frac{a_{i}}{n}$, where *a_i_* is the amino acid chemical property at position *i* from 1 to *n* |
| Window 3 | $max({\sum_{i}^{i+3} a}_{i}$), where *a_i_* is the amino acid chemical property at position *i* from 1 to *n-3* |

**Table S2: Description of AAindex1 properties selected by CLN-MLEM2 method.**

| **Property Name** | **Property Code** | **AAindex1 Description** |
| --- | --- | --- |
| C-terminal Helix Frequency | AURR980118 | Normalized positional residue frequency at helix termini C"[8] |
| Water Solvation Free Energy | CHAM820102 | Free energy of solution in water, kcal/mole[9] |
| Negative Charge | FAUJ880112 | Negative charge[10] |
| Helix Termination | FINA910104 | Helix termination parameter at position j+1[11] |
| Alpha Helix: Alpha Protein | GEIM800102 | Alpha-helix indices for alpha-proteins[12] |
| Alpha Helix: Beta Protein | GEIM800103 | Alpha-helix indices for beta-proteins[12] |
| Linker Propensity | GEOR030101 | Linker propensity from all dataset[13] |
| Thermophilic Protein Frequency | KUMS000101 | Distribution of amino acid residues in the 18 non-redundant families of thermophilic proteins[14] |
| Transmembrane Protein Frequency | NAKH900111 | Transmembrane regions of non-mt-proteins[15] |
| AH: 5^th^ Residue | QIAN880102 | Weights for alpha-helix at the window position of -5[16] |
| Beta Sheet: 6^th^ Residue | QIAN880126 | Weights for beta-sheet at the window position of 6[16] |
| Coil: 5^th^ Residue | QIAN880138 | Weights for coil at the window position of 5[16] |
| Coil: 6^th^ Residue | QIAN880139 | Weights for coil at the window position of 6[16] |
| C-terminal Frequency | RICJ880115 | Relative preference value at C-cap[17] |
| Extended No H-bond | ROBB760107 | Information measure for extended without H-bond[18] |
| Conformation State A | VASM830101 | Relative population of conformational state A[19] |
| Conformation State C | VASM830102 | Relative population of conformational state C[19] |
| Helix Free Energy | WERD780103 | Free energy change of alpha(Ri) to alpha(Rh)[20] |
| Unfolding Activation Energy | YUTK870103 | Activation Gibbs energy of unfolding, pH 7.0[21] |

## Selection Distribution of AAindex1 Properties

The distribution of each of the properties and their features among the rules selected is shown in Fig S1. The number of rules each feature occurs in is given in the colored grid for each of the rule sets (active or inactive). The total number of rules for the entire set was 236 rules. The sum of GEIM800102, the amino acid frequencies among alpha-helix indices for proteins dominated with alpha helix structure, was the most common feature selected for generating rules for describing antibacterial peptides appearing in 123 rules (52%). The window of GEIM800102 only appeared in 14 rules (6%). The AAindex1 feature QIAN880139, weights for coil at the window position of 6, had the opposite trend for rules describing active peptides. The window calculation was selected for 115 rules (49%) while its sum was only selected for two rules (1%).


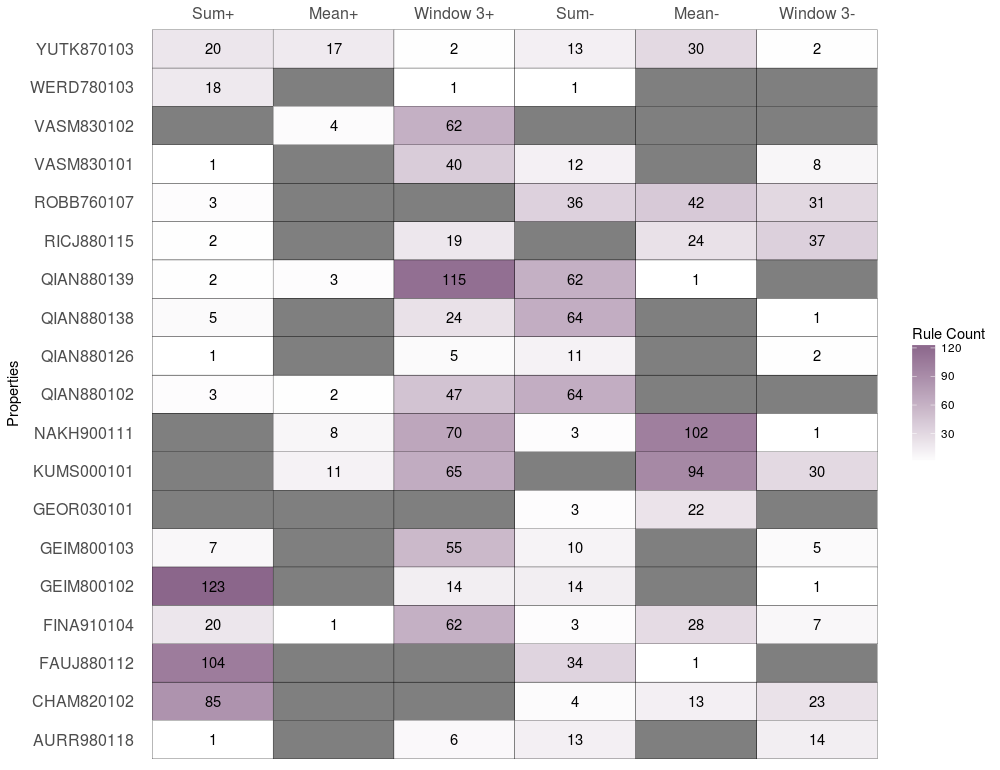


**Fig S1.** The number of rules for each of the amino acid properties selected by each calculation made. The rules are an embedded feature selection approach. The grid is colored according to the relative times each feature was selected.

No AAindex feature was used in more than 10 rules across all three calculation types for active peptides. ROBB760107, information measure for extended without H-bond, relates to the likelihood of extended conformations of the peptides. This AAindex1 feature was the only one to be selected for more and ten rules across all three calculation types for rules describing inactive peptides. This AAindex1 feature was only selected for three rules for active peptides. FINA910104 and YUTK870103 were the only AAindex1 features selected for all three calculation types for active and inactive peptide rules. YUTK870103 relates to the additive contributions of amino acids for free energy of folding at pH 7, near physiological conditions. FINA910104 relates to amino acid likelihood for being the terminal position of an α-helix.

## Antimicrobial Peptide Amino Acid Frequency and Codon Bias

We selected the standard DNA codon table to translate from polypeptide sequences to DNA codons and to reverse the translation.[22] The frequency for each amino acids was calculated as part of the antibacterial peptides in the Antimicrobial Peptide Database.[23] These frequencies were divided by standard DNA codon table frequency for the corresponding amino acid. A ratio of one means that the amino acid appears as often as would be expected by uniformly randomly selecting DNA bases and counting the resulting amino acids selected with the standard codon table. Most amino acids are within a ratio between ½ and 2, meaning that the frequency of the amino acid within antimicrobial peptides is not far from the frequency expectation from the codon table frequency alone. Three amino acids C, G and K have the largest deviations from this trend for the naturally-occurring codon table. These amino acids appear two to three times more often than their codon table frequencies would suggest.


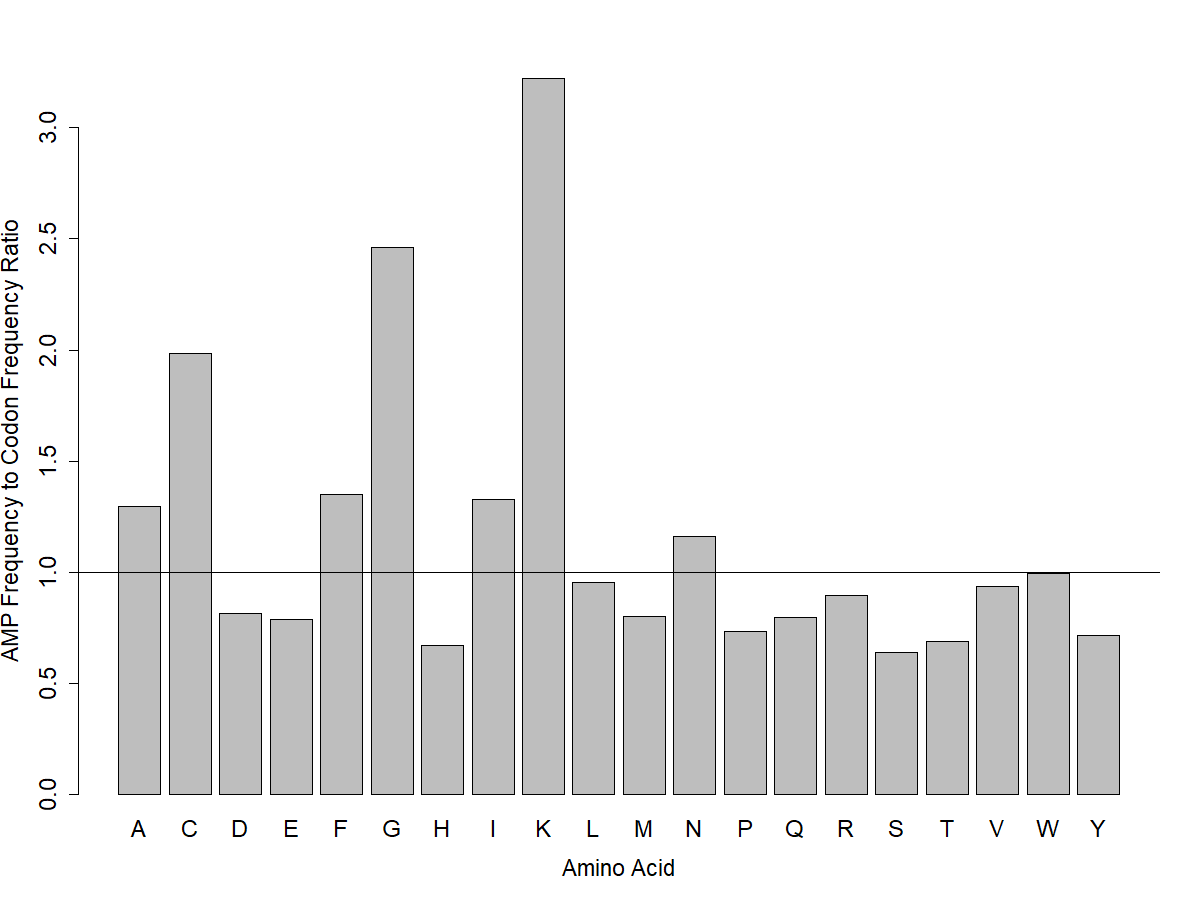


**Fig S2. Expected Antimicrobial peptide amino acid frequency by codon count.** The ratio of amino acid frequency for antibacterial AMPs from the Antimicrobial Peptide Database (APD)[23] compared to the standard DNA codon frequency for each amino acid.

## Synthesized Peptide Mass Spectra

## Hp1404 (GILGKLWEGVKSTF)


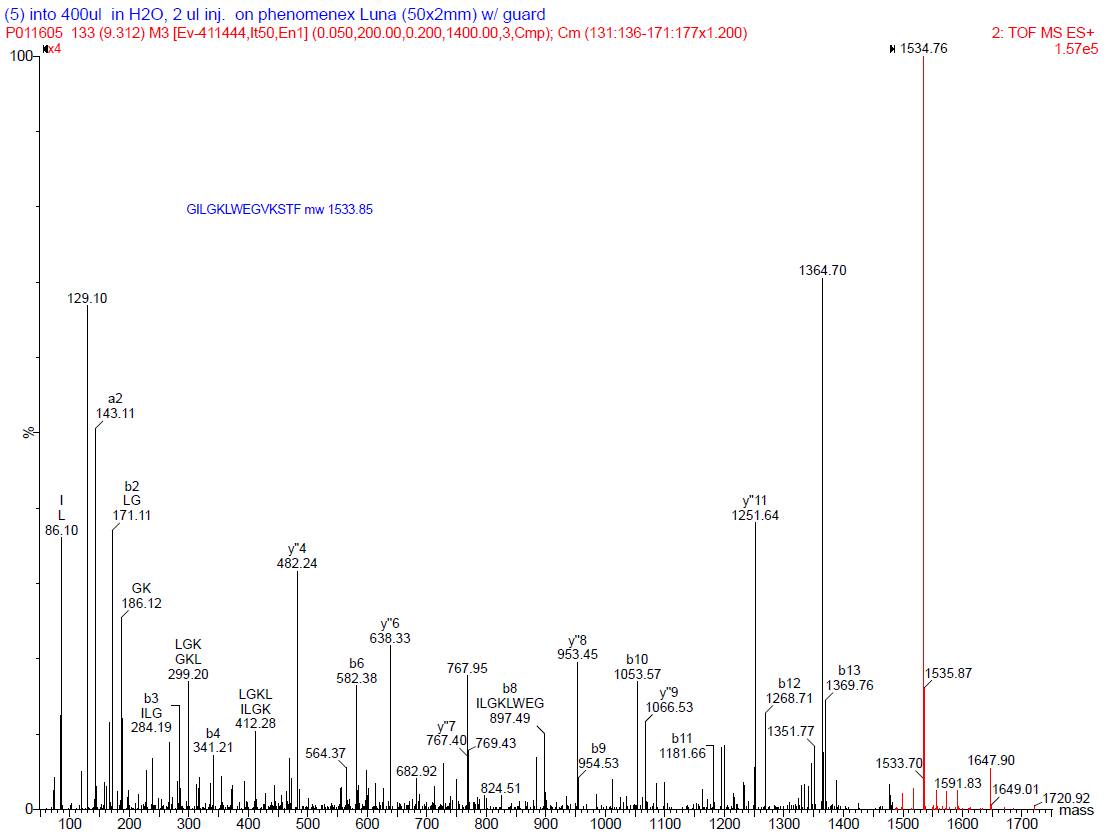


**Fig S3.** Mass spectra results for the synthesis of Hp1404.

**AMP-1 (ATLGVLWESIRGHR)**


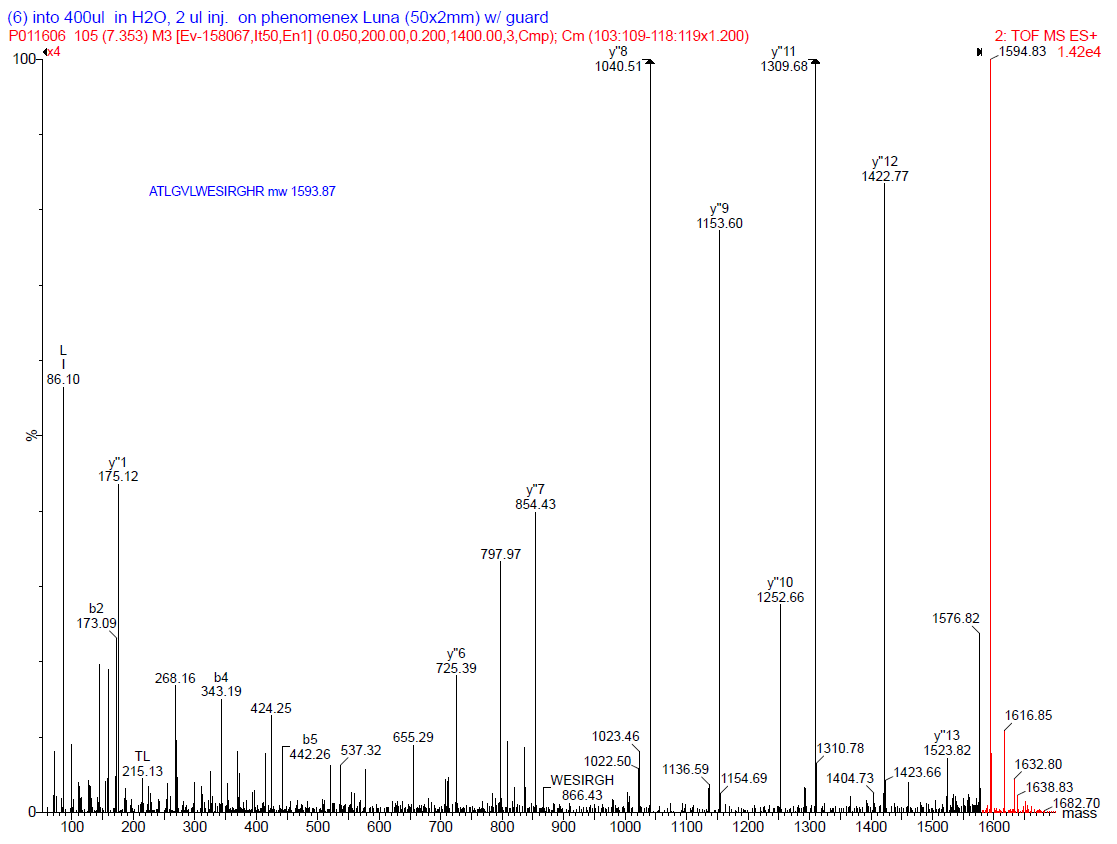


**Fig S4.** Mass spectra results for the synthesis of AMP-1.

**AMP-2 (ATLGVLWEGARGHT)**


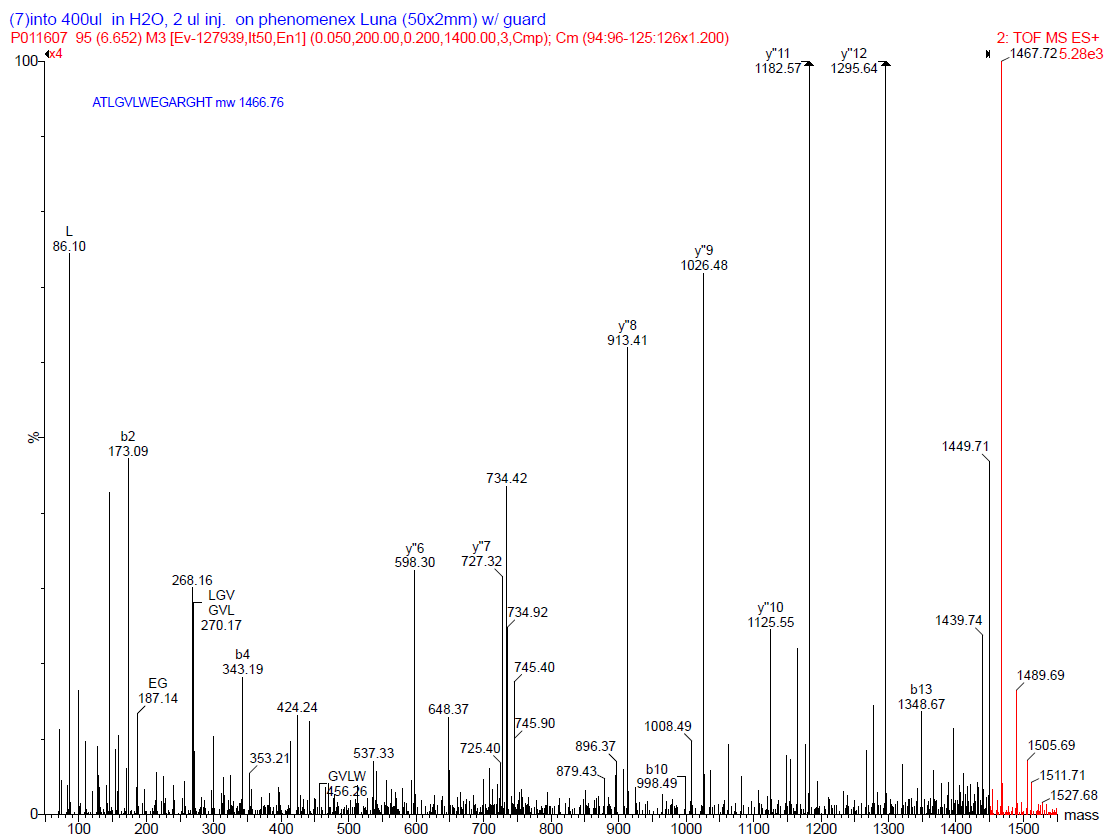


**Fig S5.** Mass spectra results for the synthesis of AMP-2.

**AMP-3 (GTLANGWEGVRTNH)**


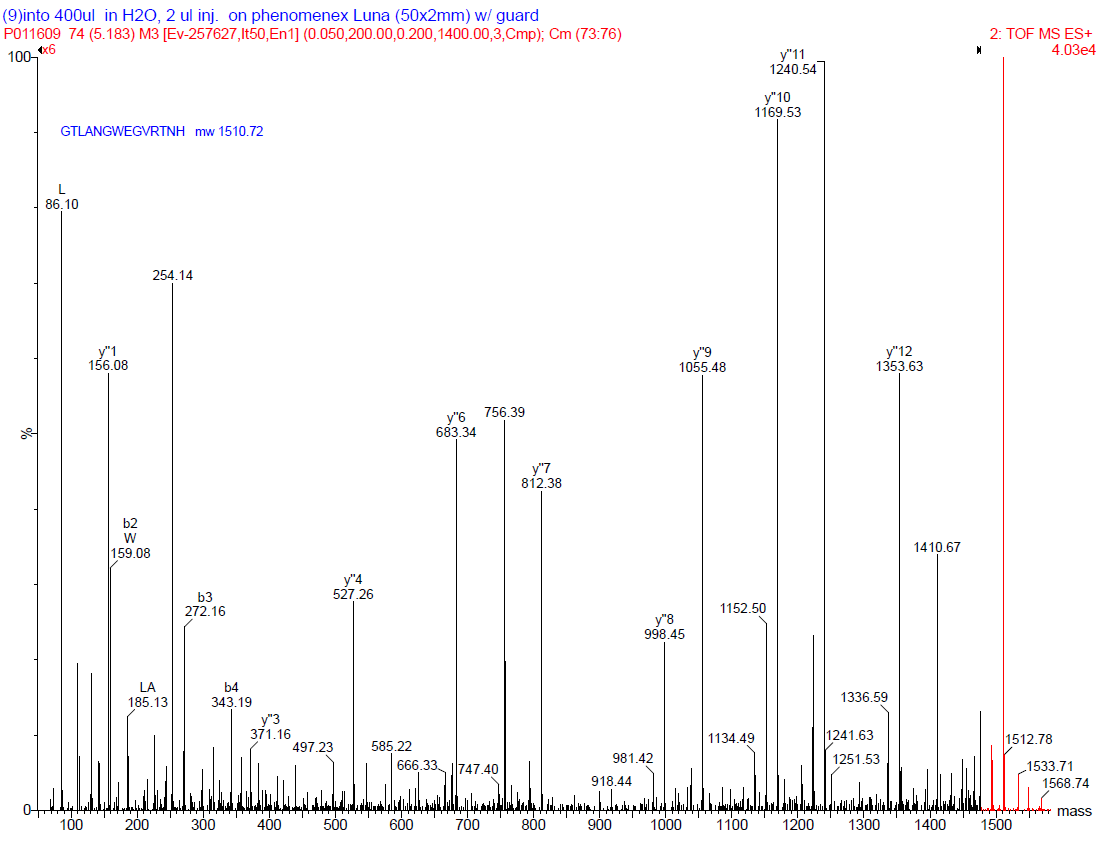


**Fig S6.** Mass spectra results for the synthesis of AMP-3.

## References

1. Xiao X, Wang P, Lin W-Z, Jia J-H, Chou K-C. **iAMP-2L: A two-level multi-label classifier for identifying antimicrobial peptides and their functional types**. *Analytical biochemistry.* 2013;**436**:168-177.

2. Wang G, Li X, Wang Z. **APD2: the updated antimicrobial peptide database and its application in peptide design**. *Nucleic Acids Research.* 2009;**37**:D933-D937.

3. Kawashima S, Kanehisa M. **AAindex: amino acid index database**. *Nucleic acids research.* 2000;**28**:374-374.

4. Charif D, Lobry JR. **SeqinR 1.0-2: A contributed package to the R project for statistical computing devoted to biological sequences retrieval and analysis**. In: *Structural approaches to sequence evolution.* Berlin, Heidelberg: Springer; 2007: 207-232.

5. Boone K, Camarda K, Spencer P, Tamerler C. **Antimicrobial peptide similarity and classification through rough set theory using physicochemical boundaries**. *BMC bioinformatics.* 2018;**19**:1-10.

6. Georgiev AG. **Interpretable numerical descriptors of amino acid space**. *Journal of Computational Biology.* 2009;**16**:703-723.

7. Veltri DP. **A computational and statistical tramework for screening novel antimicrobial peptides**. George Mason University; 2015.

8. Aurora R, Rose GD. **Helix capping**. *Protein Sci.* 1998;**7**:21-38.

9. Charton M, Charton BI. **The structural dependence of amino acid hydrophobicity parameters**. *J Theor Biol.* 1982;**99**:629-644.

10. Fauchere JL, Charton M, Kier LB, Verloop A, Pliska V. **Amino acid side chain parameters for correlation studies in biology and pharmacology**. *Int J Pept Protein Res.* 1988;**32**:269-278.

11. Finkelstein AV, Badretdinov AY, Ptitsyn OB. **Physical reasons for secondary structure stability: alpha-helices in short peptides**. *Proteins.* 1991;**10**:287-299.

12. Geisow MJ, Roberts RD. **Amino acid preferences for secondary structure vary with protein class**. *International Journal of Biological Macromolecules.* 1980;**2**:387-389.

13. George RA, Heringa J. **SnapDRAGON: a method to delineate protein structural domains from sequence data**. *J of Mol Biol.* 2002;**316**:839-851.

14. Kumar S, Tsai CJ, Nussinov R. **Factors enhancing protein thermostability**. *Protein Eng.* 2000;**13**:179-191.

15. Nakashima H, Nishikawa K, Ooi T. **Distinct character in hydrophobicity of amino acid compositions of mitochondrial proteins**. *Proteins.* 1990;**8**:173-178.

16. Qian N, Sejnowski TJ. **Predicting the secondary structure of globular proteins using neural network models**. *Journal of Molecular Biology.* 1988;**202**:865-884.

17. Richardson JS, Richardson DC. **Amino acid preferences for specific locations at the ends of alpha helices**. *Science.* 1988;**240**:1648-1652.

18. Robson B, Suzuki E. **Conformational properties of amino acid residues in globular proteins**. *J Mol Biol.* 1976;**107**:327-356.

19. Vasquez M, Nemethy G, Scheraga HA. **Computed conformational states of the 20 naturally occurring amino acid residues and of the prototype residue α-aminobutyric acid**. *Macromolecules.* 1983;**16**:1043-1049.

20. Wertz DH, Scheraga HA. **Influence of water on protein structure. An analysis of the preferences of amino acid residues for the inside or outside and for specific conformations in a protein molecule**. *Macromolecules.* 1978;**11**:9-15.

21. Yutani K, Ogasahara K, Tsujita T, Sugino Y. **Dependence of conformational stability on hydrophobicity of the amino acid residue in a series of variant proteins substituted at a unique position of tryptophan synthase alpha subunit**. *PNAS.* 1987;**84**:4441-4444.

22. Koonin EV, Novozhilov AS. **Origin and evolution of the genetic code: the universal enigma**. *IUBMB life.* 2009;**61**:99-111.

23. Wang G, Li X, Wang Z. **APD2: The updated antimicrobial peptide database and its application in peptide design**. *Nucleic Acids Res.* 2009;**37**:D933-937.
